# Real-World Evidence to Evaluate the Efficacy and Safety of Vonoprazan in Gastrointestinal Disorders in the Pakistani Population

**DOI:** 10.7759/cureus.48994

**Published:** 2023-11-18

**Authors:** Amanullah Abbasi, Shajee Ahmad Siddiqui, Bikha Ram, Jibran Umar Ayub Khan, Khalid Sheikh, Asif Ali, Waseem Raja Memon, Muhammad Rehan, Muhammad Zia ul Haq, Naresh Kumar Seetlani, Tayyab S Akhter, Masood Khoso, Asif Javed, Riaz Hussain Khokhar, Zaheer Hussain Memon, Wajid Akbar, M Naeem, Samiullah Shaikh, Abbas Khan Khattak, A. Qayoom Memon, Shaheen Bhatty, Omar Sultan, Idress Shani, Neeta Maheshwary

**Affiliations:** 1 Internal Medicine, Civil Hospital Karachi, Karachi, PAK; 2 Internal Medicine, Pakistan Institute of Medical Sciences, Islamabad, PAK; 3 Internal Medicine, Liaquat University of Medical and Health Sciences, Hyderabad, PAK; 4 Internal Medicine, Kabir Medical College, Peshawar, PAK; 5 Internal Medicine, People's University of Medical and Health Sciences for Women, Nawabshah, PAK; 6 Medicine, Dow General Hospital/Dow University of Health Sciences, Karachi, PAK; 7 Medicine, People's University of Medical and Health Sciences for Women, Nawabshah, PAK; 8 Medicine, Civil Hospital Karachi, Karachi, PAK; 9 Medicine, Sir Ganga Ram Hospital, Lahore, PAK; 10 Gastroenterology, Holy Family Hospital, Islamabad, PAK; 11 Gastroenterology, Jinnah Postgraduate Medical Center, Karachi, PAK; 12 Internal Medicine, Social Security New Born & Children Hospital (MNCH), Faisalabad, PAK; 13 Internal Medicine, Suleman Roshan Medical College, Tandoadam, PAK; 14 Internal Medicine, Bacha Khan Medical College, Peshawar, PAK; 15 Gastroenterology, Mardan Medical Complex, Peshawar, PAK; 16 Internal Medicine, Gijal Mao M/c Qasimabad, Hyderabad, PAK; 17 Gastroenterology, Abyseen Plaza Dabgari, Peshawar, PAK; 18 Gastroenterology, Private Clinic, Nawabshah, PAK; 19 Internal Medicine, Dr. Ruth K. M. Pfau Civil Hospital, Karachi, PAK; 20 Internal Medicine, Jinnah Postgraduate Medical Center, Karachi, PAK; 21 Internal Medicine, District Head Quarters (DHQ) Hospital, Faisalabad, PAK; 22 Head Medical Affairs, Helix Pharma Pvt Ltd, Karachi, PAK

**Keywords:** acid control, vonoprazan, dyspepsia, helicobacter pylori, gastroesophageal reflux disease

## Abstract

Background and aim: While proton pump inhibitor (PPI) therapy has proven to be effective in managing gastroesophageal reflux disease (GERD), a notable portion of patients who experience GERD symptoms may not respond to this treatment. Research suggests that roughly 30% of individuals with a presumed GERD diagnosis may continue to experience symptoms, whether partially or completely, even when receiving PPI therapy. The aim of this study was to assess the treatment of gastrointestinal diseases with a novel potassium-competitive acid blocker (P-CAB), vonoprazan, in terms of its effectiveness and safety in the Pakistani population.

Methods: This prospective, multicenter, observational study was conducted in Pakistan. This study included 1,642 patients from January 2023 to August 2023, aged 18 years, with gastrointestinal disorders. All demographic data, medical history, GERD severity assessment questionnaire (GerdQ), and laboratory parameters, including stool assessment for Helicobacter pylori (H. pylori), were observed. Patients were orally treated with vonoprazan at doses of 10 mg or 20 mg, once or twice daily. Statistical analysis was done by one-way ANOVA.

Results: Out of 1,642 patients, 840 (51.2%) were males and 802 (48.8%) were females, with a mean age of 39.81±14.61 years. The mean GerdQ score at baseline was 20.37±15.87, 7.24±8.15 at the second week of treatment, and 3.70±6.31 at the fourth week of treatment (p<0.001). 90.74% of patients achieved H. pylori eradication. Most patients were acid regurgitation and heartburn-free for >70% of days. Most of the patients, 1,283 (78.13%), exhibited good treatment compliance. Mild adverse events were reported in 37 (2.3%) patients.

Conclusions: The use of vonoprazan significantly reduced the likelihood of GERD by improving symptoms and was also highly effective in the elimination of H. pylori infections. Vonoprazan was generally well tolerated.

## Introduction

Gastroesophageal reflux disease (GERD) is a chronic illness of the gastrointestinal tract in which the contents of the stomach move up in the esophagus, most commonly due to a decreased lower esophageal tone, resulting in troublesome symptoms. In the United States (US), it is the most frequently prevalent digestive disease, with a reported prevalence rate of about 20%, causing substantial financial encumbrance and badly disrupting the quality of life [1,2]. Chronic gastritis and severe gastro-duodenal pathologies such as peptic ulcer disease (PUD), stomach cancer, and mucosal lymphoid tissue lymphoma are associated with Helicobacter pylori (H. pylori) [3]. Rather than actual data, estimates are usually used for assessing the incidence of GERD around the world [4]. 12% of white, 3% African American, and 2% Asian patients had severe GERD, according to research that examined endoscopic results from over 2,600 consecutive individuals [5].

GERD is caused by a combination of internal and structural factors, which mainly include the destruction of the esophagogastric sphincter and exposure of the esophagus to the acidic fluids of the stomach. Typically, symptoms of GERD clinically manifest as heartburn and regurgitation. Atypically, GERD can present as extraesophageal symptoms like asthma, chronic cough, dental erosions, hoarseness of voice, and throat irritation [6,7]. Endoscopic and histopathological findings revealed that GERD is categorized into three dissimilar phenotypes: erosive esophagitis (EE), non-erosive reflux disease (NERD), and Barrett esophagus (BE) [8]. The most predominant phenotype is NERD, which is observed in 60%-70% of cases; subsequently, EE is found in 30% of cases; and lastly, BE is observed in 6%-12% of patients with GERD [1,8,9].

The occurrence of GERD is marginally higher in men than women [10]. NERD is the most prevalent phenotype in women with GERD symptoms, whereas EE is probably more prevalent in males [11]. Nonetheless, male patients who have severe GERD symptoms have a greater prevalence of BE (23%) than female patients (14%) [12].

Presently, there is ambiguity to explicate the development of GERD. Over the past few years, numerous risk factors have been identified and linked to GERD. The pathophysiology of GERD begins with impaired motor irregularities; for example, the impaired muscular activity of the esophagus reduces esophageal acid clearance, impaired LES function, transient LES relaxation, and delays in the clearance of stomach contents [13]. Structural aspects such as the existence of a hiatal hernia or elevated intra-abdominal pressure due to fatness or pregnancy are risk factors for the development of GERD [13]. Similarly, one meta-analysis done by Hampel et al. showed that obesity raised the risk of GERD, EE, and esophageal cancer [14]. Furthermore, various risk factors are independently linked with GERD symptoms, including age over 50 years, poor socioeconomic status, tobacco or alcohol addiction, connective tissue diseases, pregnancy, postprandial supination, and overuse of medicines, including anticholinergic drugs, non-steroidal anti-inflammatory drugs (NSAID), benzodiazepines, albuterol, nitroglycerin, calcium channel blockers, anti-depressants, and glucagon [15,16].

Diagnosis of GERD depends on the patient's presenting complaints or in combination with further factors like a positive response to anti-secretory treatment, esophagogastroduodenoscopy (EGD), and ambulatory reflux observation. GERD can be presumably detected in most patients based on their presenting complaints of indigestion and regurgitation [17]. In the absence of alarming symptoms such as a family history of EC in first-degree relatives, dysphagia, anemia, odynophagia, loss of weight, and hematemesis, patients can receive empirical therapy with proton pump inhibitors (PPIs) with no need for further investigations, and responsiveness to therapy confirms the GERD [17]. Conversely, another meta-analysis by Numan et al. negated the efficacy of this empirical PPI investigative therapy [18]. Consequently, patients who exhibit both the usual symptoms of GERD and any alarming symptoms must undergo an EGD to exclude complications of GERD. The complications of GERD are EE, BE, peptic stricture, and esophageal carcinoma. American College of Gastroenterology (ACG) guidelines recommend that distal esophageal biopsies are not usually suggested for the detection of GERD [17].

The main objective of managing GERD is to resolve its symptoms and prevent its complications. Therefore, treatment possibilities include lifestyle modifications, treatment with antacids and anti-secretory drugs, surgical treatments, and endoluminal therapies. Over the past years, lifestyle modifications along with PPIs have been considered mainstays in the management of GERD. Losing weight is also essential for lowering the chance of developing GERD [19]. In cases of unresponsiveness to lifestyle modifications, medical therapy is recommended for patients with GERD. PPI-based triple therapy, comprising PPI, amoxicillin, clarithromycin, or metronidazole, is recommended for H. pylori infections [20,21]. However, due to the development of increased H. pylori resistance to clarithromycin and metronidazole, the efficacy of PPI-based triple therapy has decreased over the past decade [22]. In a study conducted in China, clarithromycin resistance rates for H. pylori were found at 63.4%, and metronidazole resistance rates at 52.6% [23]. While PPI therapy has proven to be effective in managing GERD, a notable portion of patients who experience GERD symptoms may not respond to this treatment. Research suggests that roughly 30% of individuals with a presumed GERD diagnosis may continue to experience symptoms, whether partially or completely, even when receiving PPI therapy. One of the most frequent scenarios encountered during outpatient visits to gastroenterology offices is patients presenting with unresolved symptoms after PPI therapy has failed to provide relief [24].

Various strategies have been proposed to enhance the successful eradication of H. pylori infection. These approaches encompass a range of options, including therapies containing bismuth and those without it (both quadruple therapies), such as sequential, concomitant, hybrid, and reverse hybrid therapies. Additionally, high-dose dual therapy and vonoprazan-based triple therapy have been suggested to improve treatment outcomes [25]. For individuals receiving their initial H. pylori eradication treatment, several regimens are available for consideration. These options include a 14-day standard triple therapy, a non-bismuth quadruple therapy, a seven-day standard triple therapy following a clarithromycin resistance test, and a bismuth-based quadruple therapy [26].

Vonoprazan is a novel oral acid-suppressing medication that is a class of H+-K+ ATPase inhibitors similar to PPIs. In contrast to PPIs, Vonoprazan is a reversible H+-K+ ATPase inhibitor [27]. In Japan since 2015, Vonoprazan has been accepted to treat H. pylori infection, gastro-duodenal ulcer, and reflux esophagitis. It has been proven by several clinical trials that the acid-inhibitory effects of vonoprazan, which is 350 times more potent than PPIs, are stronger, quicker, and last longer than those of PPIs [28-30]. Consequently, the elimination rate of H. pylori by vonoprazan was improved as compared to PPIs. Various meta-analyses have recently concluded that vonoprazan-containing triple therapy outperforms PPI-containing triple therapy [31-33].

Vonoprazan maintains its resistance to changes in pH levels, making it suitable for use in both laboratory and clinical settings, even in situations with either neutral or highly acidic pH conditions. In a study examining its effectiveness for treating GERD at the four-week mark, different doses of vonoprazan (5, 10, 20, and 40 mg) were compared to 30 mg of lansoprazole. The results showed healing rates of 92.3%, 92.5%, 94.4%, 97.0%, and 93.2%, respectively. Vonoprazan demonstrated notably higher efficacy, achieving a 97.7% rate of ulcer constriction. In a randomized trial involving 141 patients with a history of H. pylori infection, the vonoprazan group showed significantly superior results, with an eradication rate of 95.8% using the vonoprazan 20 mg, AMX 750 mg, and CLB 200-400 mg regimen. Among patients (n = 7) who received vonoprazan therapy, complete healing of the gastric mucosa was observed in 87.5% of cases. In a randomized, double-blind study involving 650 individuals with gastric or duodenal ulcers, 641 subjects received initial therapy, resulting in a first-line eradication rate of 92.6% with vonoprazan. A randomized controlled trial was conducted with 2,715 patients aged 63 and older who were administered vonoprazan and compared to the traditional PPI regimen. There were 10 reported instances of diarrhea, a known side effect that was also observed with conventional PPI use. In another randomized controlled trial, H. pylori eradication rates were 91.4% with vonoprazan and 74.5% with PPIs. The first group experienced adverse events in 32.7% of cases, while the second group had a 40.5% incidence, suggesting that vonoprazan is more effective with fewer side effects compared to PPIs [34].

In Pakistan, in order to consider the typical environment, type of food, and lifestyle of our population, it is imperative to evaluate the efficacy and safety of vonoprazan in an indigenous setting. Therefore, this study was intended to assess the efficacy and safety of vonoprazan therapy in the management of gastro-esophageal disorders. The research question of this study was “Does the use of vonoprazan as a novel potassium-competitive acid blocker (P-CAB) in the treatment of gastrointestinal diseases effectively improve symptoms and eliminate H. pylori infections in the Pakistani population?” The null hypothesis (H0) suggests that the use of vonoprazan for the treatment of gastrointestinal diseases in the Pakistani population does not lead to a significant improvement in symptoms or the eradication of H. pylori infections. Conversely, the alternate hypothesis (H1) suggests that vonoprazan treatment significantly improves symptoms and results in the eradication of H. pylori infections in this population.

## Materials and methods

This is a six-week prospective, multicenter, observational study. The study was conducted at 25 sites (Karachi, Lahore, Islamabad, Faisalabad, Nawabshah, Hyderabad, Peshawar, and Rawalpindi) in Pakistan from January 2023 to August 2023 after receiving ethics committee approval from the Ethical Review Board of the Liaquat College of Medicine and Dentistry (LCMD). This study included 1,642 patients with gastric conditions (including GERD, EE, Gastritis, dyspepsia associated with H. pylori, peptic ulcer disease (PUD), ulcers induced by prolonged use of an NSAID, etc.). Whereas the presence of clinically significant neurological, cardiovascular, hepatic, and renal dysfunction, immune system-related disease, pregnant and lactating mothers, alcoholics and drug abusers, known allergies to the study drug Vonoprazan (VPN) manufactured by Helix Pharma Pvt Ltd, and patients who failed to comply with follow-up were excluded from the study.

All patients gave written, informed consent before assessments or procedures. All demographic data, medical history, GERD severity assessment questionnaire, and laboratory parameters, including the patient’s stool assessment for H. pylori infection, were documented. The GerdQ is a brief, certified self-assessment questionnaire that assesses the presence of GERD and how symptoms affect patients' everyday lives. A baseline visit, a treatment phase, and a follow-up period made up the protocol. According to the physician’s decision, the treatment was initiated and was considered the first visit as soon as the patient administered the first dose of vonoprazan treatment until the end of therapy. Treatment with vonoprazan began in doses of 10 and 20 mg once or twice daily, depending on the condition and severity of symptoms. All the patients included in this study were evaluated at baseline, at the second and fourth weeks of the treatment. Patients’ data on the safety and efficacy profile of vonoprazan medication was gathered and assessed. Statistical data was analyzed using SPSS version 23.0 (IBM Corp., Armonk, NY). A statistical method, one-way ANOVA, was employed to determine the association between the means of the GerdQ score and efficacy. A p-value of less than 0.05 was considered statistically significant throughout the study.

## Results

A total of 1642 patients with diagnosed gastric disorders were enrolled in this study, of whom 840 (51.2%) were males and 802 (48.8%) were females, with a mean age of 39.81±14.61 years. Among 1642 patients, about 376 (22.9%) used NSAIDs for longer durations. The history of addiction revealed that 147 (8.95%) patients were smokers and five (0.3%) were tobacco addicts. Concerning diagnosis, 559 (34.04%) patients were diagnosed with GERD, gastritis was diagnosed in 446 (27.16%) patients, and EE was diagnosed in 88 (5.3%) patients. Among all, 735 (44.76%) patients were H. pylori positive. GERD was diagnosed in 424 (25.7%) patients. Two hundred sixty (15.8%) patients were diagnosed with dyspepsia; gastric ulcers induced by NSAIDs were diagnosed in 37 (2.25%) patients; and PUD was diagnosed in 22 (1.34%) patients as shown in Table 1.

**Table 1:** Demographic characteristics of patients. (n=1642)

Variable			Mean±SD n(%)
Gender (n=1642)	Male		840 (51.2%)
	Female		802 (48.8%)
Age (n=1642)			39.81±14.61
Long term NSAID use (n=1642)		Yes	376 (22.9%)
		No	1266 (77.1%)
History of Addiction (n=1642)		Smoker	147 (8.95%)
		Tobacco	5 (0.3%)
		Nil	1490 (90.74%)
Diagnosis (n=1642)		H. pylori	735 (44.76%)
		GERD	424 (25.8%)
		Erosive Esophagitis	28 (1.7%)
		Gastritis	136 (8.3%)
		Dyspepsia	260 (15.8%)
		NSAIDs induced Ulcers	37 (2.25%)
		Peptic ulcer disease PUD	22 (1.34%)
Family History of EC in first degree relatives			481 (29.3%)

The mean GerdQ score at baseline was 20.37±15.87 of all patients, 7.24±8.15 after the second week of 1455 patients, and 3.70±6.31 after the fourth week of 1620 patients. Comparisons of the GerdQ Score revealed a significant difference observed between the means of the GerdQ Score at baseline (20.37±15.87) and second week (7.24±8.15) (p<0.001). Furthermore, a significant difference (p<0.001) was observed between the means of the GerdQ score at baseline (20.37±15.87) and the fourth week (3.70±6.31). Moreover, a significant difference was also observed between the means of the GerdQ score at the second (7.24±8.15) and fourth week (3.70±6.31) assessments (p<0.001) (Table 2).

**Table 2:** Comparison of GerdQ Score at baseline, second and fourth week (n=1,642)

Variables	Mean ± SD	P-value
GerdQ Score at Baseline	20.37±15.87	<0.001
GerdQ Score at 2^nd^ Week	7.24±8.15	
GerdQ Score at Baseline	20.37±15.87	<0.001
GerdQ Score at 4^th^ Week	3.70±6.31	
GerdQ Score at 2^nd^ Week	7.24±8.15	<0.001
GerdQ Score at 4^th^ Week	3.70±6.31	

The GerdQ Score at baseline revealed that positive predictors such as regurgitation, heartburn, sleep disturbances, and acid suppressor use scored 3 (the highest score) from four to seven days in 705 (42.94%) patients in terms of heartburn. Similarly, most of the patients 546 (33.25%) reported a score of 3 from four to seven days in terms of regurgitation, and 381 (23.20%) showed sleep disturbances and scored 3 from four to seven days. In terms of the use of acid-suppressing medication, a relatively small number of patients 210 (12.79%) reported scores of 3 from four to seven days. On the other hand, negative predictors that address dyspeptic symptoms such as pain and nausea were less frequently reported 271 (16.50%) and 291 (17.72%) in patients, respectively, that scored 3 at day 0.

The GerdQ Score at the second week revealed that score 1 was reported at day 1 and observed most frequently in 452 (42.94%) patients in terms of heartburn. Most of the patients 515 (33.25%) reported a score of 1 on day 1 in terms of regurgitation. Beside this, only 260 (17.87%) patients showed sleep disturbances at four to seven days and scored 3, and only 252 (17.32%) patients reported a score of 3 from four to seven days with the use of acid-suppressing medication. On the other hand, negative predictors that address dyspeptic symptoms such as pain were more frequently reported in 581 (30.93%) patients at day 1, and nausea was more frequently reported in 494 (33.95%) patients at day 1.

The GerdQ Score at the fourth week revealed that most of the patients 532 (32.84%) scored 0 at day 0 in terms of heartburn and 549 (33.89%) in terms of regurgitation. Furthermore, 659 (40.68%) of patients who scored 0 reported fewer sleep disturbances on day 0, and 744 (45.93%) reduced their use of acid-suppressing medications. On the other hand, negative predictors that address dyspeptic symptoms such as pain were more frequently reported in 560 (34.57%) patients at day 0, and nausea was also frequently reported in 574 (35.43%) patients at day 0.

**Figure 1:**
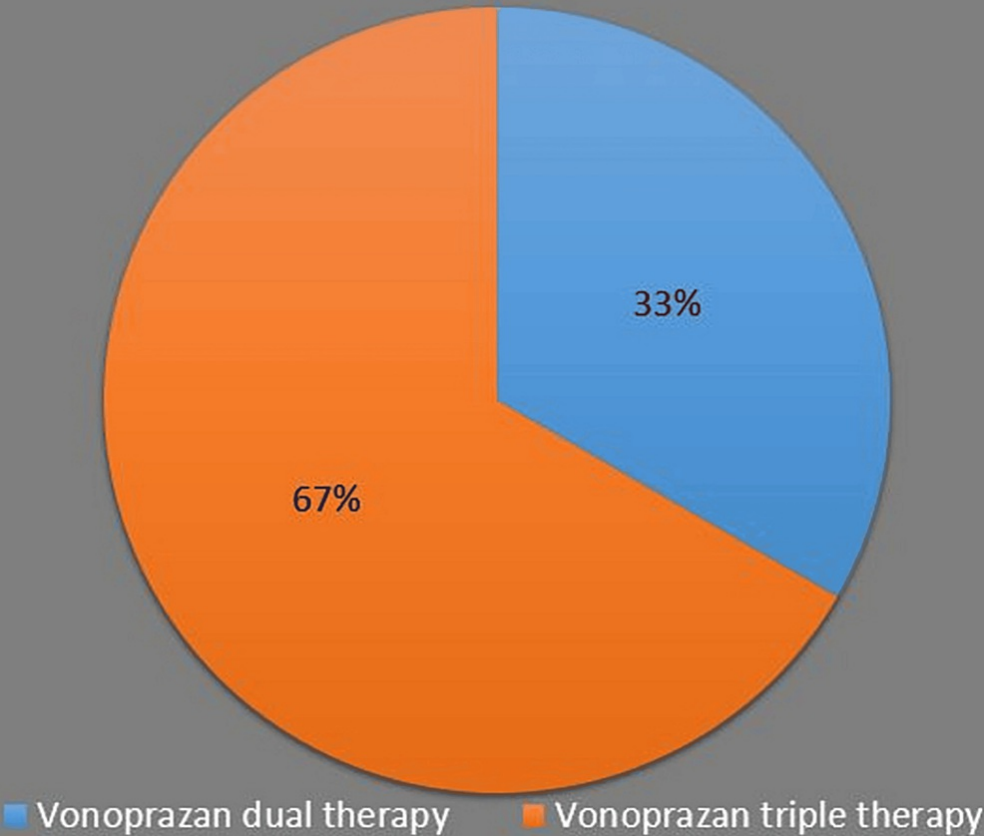
Eradication of H. pylori rate with drug regimens of vonoprazan (n=735)

In gastrointestinal disorders except H. pylori, 10 mg OD in 90 (5.48%) patients, 10mg BD in 20 (1.23%) patients, 20 mg OD in 752 (45.79%) patients, and 20 mg BD in 45 (2.74%) patients were prescribed. A standard regimen dose of 20 mg BD was used in 735 (44.76%) H. pylori patients. Vonoprazan treatment in 735 H. pylori-positive patients revealed that a dual regimen along with vonoprazan was used in 245 (33.33%) patients, and a triple regimen combined with vonoprazan was used in 490 (66.66%) patients, as shown in Figure 1. The H. pylori assessment was positive at baseline in 735 (44.8%) patients. H. pylori remained positive in 68 (4.2%) patients at the end of the fourth week. 90.74% of patients achieved H. pylori eradication.

**Figure 2:**
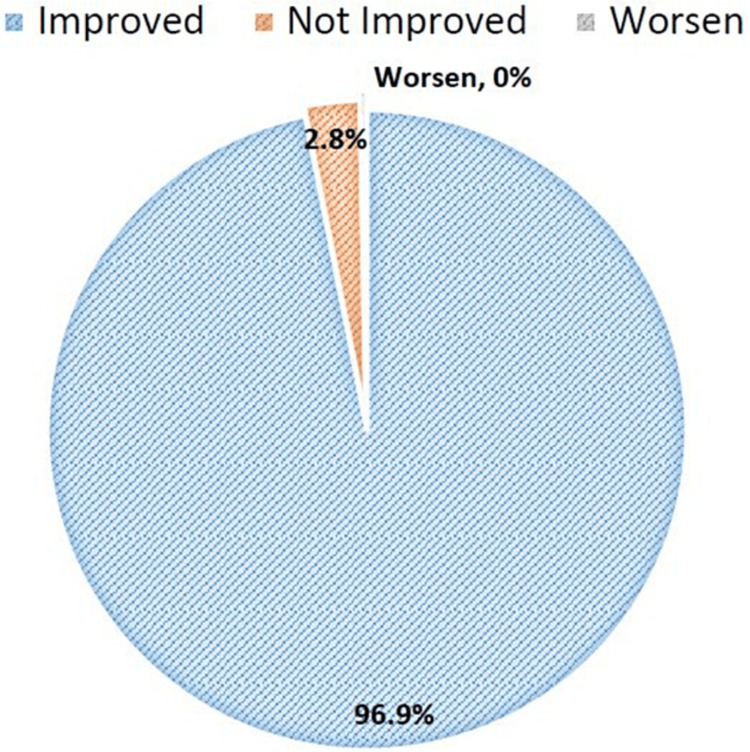
Overall improvement in condition after treatment with vonoprazan (n=1,620)

Overall, most patients 1,570 (96.9%) had improved overall condition by the end of the study, with only 45 (2.8%) patients reporting persistent symptoms and 0.3% worsen as depicted in Figure 2.

Table 3 provides an overview of the adverse events observed during the study period. The table lists various side effects, along with the number of occurrences n (%). Among the reported side effects, abdominal pain was observed in 2 cases (0.12%), palpitations and headaches each occurred in two and four cases (0.12% and 0.24%, respectively). Loose motion was reported in 17 instances (1.04%), nausea and vomiting occurred in 12 cases (0.7%).

**Table 3:** Overview of adverse events during the study period

Variable		n(%)
Side Effects (n=1620)	Abdominal pain	2 (0.12)
	Palpitation	2 (0.12)
	Headache	4 (0.24)
	Loose motion	17 (1.04)
	Nausea and vomiting	12 (0.7)

## Discussion

PPIs are considered the first line of treatment for GERD, but many studies have reported that some patients with mucosal injury of the esophagus do not respond and that many patients with GERD are resistant to or only partially respond to PPI treatment. Therefore, recent trials compared 20 mg of vonoprazan once daily to PPIs in terms of effectiveness, safety, and side effects and reported improved vonoprazan response [35]. Similarly, this study also showed that vonoprazan is effective and tolerable for treatment in terms of reducing the burden of H. pylori infection and reducing the symptoms of GERD [36].

There are many gastrointestinal conditions, including GERD, that are affected by H. pylori infection, although its contribution to the development of GERD is still debatable [37]. Furthermore, the majority of studies investigating the relationship between H. pylori infection and GERD have shown no causative link [38,39]. Moreover, esophagitis is thought to be the primary cause of acid interactions with the esophageal mucosa [40]. However, these study results support H. pylori's involvement in gastric diseases. The study reported that about 735 (44.8%) patients had positive results in an H. pylori stool test at baseline, indicating H. pylori was the main causative microorganism for developing gastric disorders.

Clinically, it can be exceedingly difficult to manage GERD patients who are resistant to PPIs. According to one study, people with PPI-resistant GERD who switched to vonoprazan medication showed an improvement in GERD symptoms in addition to fewer side effects. Eighteen patients (69.2%) reported improved symptoms following the switch from PPI to vonoprazan medication, whereas six (23.1%) showed no change and two (7.7%) had worsening symptoms [35]. These findings are comparable with the findings of the present study, which stated that 1570 (96.9%) patients had overall symptoms that improved, whereas 45 patients (2.8%) had no change, and five (0.3%) had symptoms that got worse after the use of vonoprazan treatment. In a similar manner, another study compared the long-term safety and effectiveness of vonoprazan and lansoprazole in individuals with EE. Their investigation reported that vonoprazan was safe and efficient during long-term maintenance [41]. The present study also confirmed that vonoprazan at 10 or 20 mg at the end of the fourth week was safe and tolerable in patients with gastric disorders, lowering the GerdQ score.

Likewise, another retrospective study also noticed vonoprazan's effects in patients with PPI-resistant GERD. Among the 24 patients who were receiving vonoprazan 10 mg daily for their PPI-resistant GERD. The Izumo scale score was considerably lower after one month of vonoprazan therapy (before 5.8±1.7, at one month 1.9±1.9, p<0.001), and the overall improvement rates of GERD symptoms were 88% (21 out of 24 patients) and 42% (10 out of 24 patients), respectively, with no patient reporting any negative outcome. In conclusion, individuals with GERD who are resistant to PPIs respond well to vonoprazan 10 mg daily [42]. These findings were corroborated with the present study and showed that overall symptoms were improved in about 1,570 (96.9%) patients at the end of the fourth week with negligible side effects. Moreover, the mean GERD score was substantially reduced from baseline (20.37±15.87) to week 4 (3.70±6.31) (p<0.001).

Another study conducted on Pakistani GERD patients reported that smoking and regular use of NSAIDs were significant risk factors for GERD. The two main problems that patients had during the previous year were dyspepsia and a history of GERD symptoms, and 84.38% of patients were experiencing dyspepsia. Patients also reported psychological symptoms like nervousness, agitation, and hallucinations, alone or in combination. This may be a result of the patients' ongoing psychological suffering [43]. An Iranian study showed that GERD patients frequently had headaches, mental distress, tension, nightmares, and agitation. These symptoms were also frequently associated with other GERD symptoms, such as nervousness. In 231 cases (24.21%), a familial past history of acid peptic disorder was also reported in this study. Another study in the Saudi population observed a link between GERD symptoms and a family history of gastrointestinal diseases. However, the present study contradicted the previously mentioned studies and found that most patients reporting GERD did not have a history of smoking (90.74%) or long-term NSAID usage (77.1%). In contrast, 260 (15.8%) patients reported having a history of dyspepsia.

The adverse events observed during the study, included abdominal pain, palpitations, headaches, loose motion, and nausea and vomiting. Abdominal pain was the least common, while loose motion had the highest incidence. Zuberi et al.'s study revealed a reduced occurrence of adverse effects and a higher level of tolerability observed in vonoprazan-based dual therapy when compared to the conventional triple therapy. In the vonoprazan group, only a few patients experienced mild symptoms like nausea, vomiting, bloating, and diarrhea, and none of these instances were severe enough to necessitate the discontinuation of the treatment regimen [44]. A meta-analysis comparing the efficacy and safety of vonoprazan-based triple therapy to PPI-based triple therapy for the eradication of H. pylori. This meta-analysis demonstrated a significantly lower incidence of adverse effects and better tolerability in the vonoprazan-based triple therapy group compared to the PPI-based triple therapy group [45].

In this study, the research was conducted over a relatively short duration of six weeks, and it featured extensive exclusion criteria. Notably, the study focused exclusively on the vonoprazan treatment group, lacking a comparative control group, which limits the depth of the analysis. Future research endeavors should consider longer-term investigations, incorporate comparative analyses, and ensure the inclusion of diverse patient populations. It is essential to provide more comprehensive information about adverse events and consider conducting subgroup analyses to explore the specific impacts of vonoprazan on different gastric conditions.

## Conclusions

This study concluded that the use of vonoprazan treatment significantly reduced the likelihood of GERD by improving the symptoms from baseline to the end of the fourth week of treatment. Furthermore, vonoprazan therapy was quite successful in eradicating H. pylori. Vonoprazan also had a high safety profile and was well-accepted by patients with gastrointestinal conditions. Therefore, avoiding the consequences of GERD requires early detection of symptoms and management with appropriate therapy, which has high safety and is more effective for the management of the gastric acid disorder.
