# Correction: Real-World Evidence to Evaluate the Efficacy and Safety of Vonoprazan in Gastrointestinal Disorders in the Pakistani Population

**DOI:** 10.7759/cureus.c147

**Published:** 2023-12-06

**Authors:** Amanullah Abbasi, Shajee Ahmad Siddiqui, Bikha Ram, Jibran Umar Ayub Khan, Khalid Sheikh, Asif Ali, Waseem Raja Memon, Muhammad Rehan, Muhammad Zia ul Haq, Naresh Kumar Seetlani, Tayyab S Akhter, Masood Khoso, Asif Javed, Riaz Hussain Khokhar, Zaheer Hussain Memon, Wajid Akbar, M Naeem, Samiullah Shaikh, Abbas Khan Khattak, A. Qayoom Memon, Shaheen Bhatty, Omar Sultan, Idress Shani, Neeta Maheshwary

**Affiliations:** 1 Internal Medicine, Civil Hospital Karachi, Karachi, PAK; 2 Internal Medicine, Pakistan Institute of Medical Sciences, Islamabad, PAK; 3 Internal Medicine, Liaquat University of Medical and Health Sciences, Hyderabad, PAK; 4 Internal Medicine, Kabir Medical College, Peshawar, PAK; 5 Internal Medicine, People's University of Medical and Health Sciences for Women, Nawabshah, PAK; 6 Medicine, Dow General Hospital/Dow University of Health Sciences, Karachi, PAK; 7 Medicine, People's University of Medical and Health Sciences for Women, Nawabshah, PAK; 8 Medicine, Civil Hospital Karachi, Karachi, PAK; 9 Medicine, Sir Ganga Ram Hospital, Lahore, PAK; 10 Gastroenterology, Holy Family Hospital, Islamabad, PAK; 11 Gastroenterology, Jinnah Postgraduate Medical Center, Karachi, PAK; 12 Internal Medicine, Social Security New Born & Children Hospital (MNCH), Faisalabad, PAK; 13 Internal Medicine, Suleman Roshan Medical College, Tandoadam, PAK; 14 Internal Medicine, Bacha Khan Medical College, Peshawar, PAK; 15 Gastroenterology, Mardan Medical Complex, Peshawar, PAK; 16 Internal Medicine, Gijal Mao M/c Qasimabad, Hyderabad, PAK; 17 Gastroenterology, Abyseen Plaza Dabgari, Peshawar, PAK; 18 Gastroenterology, Private Clinic, Nawabshah, PAK; 19 Internal Medicine, Dr. Ruth K. M. Pfau Civil Hospital, Karachi, PAK; 20 Internal Medicine, Jinnah Postgraduate Medical Center, Karachi, PAK; 21 Internal Medicine, District Head Quarters (DHQ) Hospital, Faisalabad, PAK; 22 Head Medical Affairs, Helix Pharma Pvt Ltd, Karachi, PAK

This article has been corrected at the request of the author to update the last author's affiliation and add manufacturer information regarding the study drug Vonoprazan to the first paragraph in the Materials and Methods section. The revised sentence now reads as follows: "Whereas the presence of clinically significant neurological, cardiovascular, hepatic, and renal dysfunction, immune system-related disease, pregnant and lactating mothers, alcoholics and drug abusers, known allergies to the study drug Vonoprazan (VPN) manufactured by Helix Pharma Pvt Ltd, and patients who failed to comply with follow-up were excluded from the study."

